# Isolation of Human Monoclonal Antibodies to the Envelope E2 Protein of Hepatitis C Virus and Their Characterization

**DOI:** 10.1371/journal.pone.0055874

**Published:** 2013-02-07

**Authors:** Yohko K. Shimizu, Minako Hijikata, Masamichi Oshima, Kazufumi Shimizu, Harvey J. Alter, Robert H. Purcell, Hiroshi Yoshikura, Hak Hotta

**Affiliations:** 1 Division of Microbiology, Center for Infectious Diseases, Kobe University Graduate School of Medicine, Kobe, Japan; 2 Department of Respiratory Diseases, Research Institute, National Center for Global Health and Medicine, Tokyo, Japan; 3 Department of Immunology, National Institute of Infectious Diseases, Tokyo, Japan; 4 Department of Gynecology, Nihon University School of Medicine, Tokyo, Japan; 5 Department of Transfusion Medicine, Warren Grant Magnuson Clinical Center, National Institutes of Health, Bethesda, Maryland, United States of America; 6 Laboratory of Infectious Diseases, National Institute of Allergy and Infectious Diseases, National Institutes of Health, Bethesda, Maryland, United States of America; 7 Food Safety Division, Ministry of Health Labor and Welfare, Tokyo, Japan; The Scripps Research Institute and Sorrento Therapeutics, Inc., United States of America

## Abstract

We isolated and characterized two human monoclonal antibodies to the envelope E2 protein of hepatitis C virus (HCV). Lymphoblastoid cell lines stably producing antibodies were obtained by immortalizing peripheral blood mononuclear cells of a patient with chronic hepatitis C using Epstein-Barr virus. Screening for antibody-positive clones was carried out by immunofluorescence with Huh7 cells expressing the E2 protein of HCV strain H (genotype 1a) isolated from the same patient. Isotype of resulting antibodies, #37 and #55, was IgG1/kappa and IgG1/lambda, respectively. Epitope mapping revealed that #37 and #55 recognize conformational epitopes spanning amino acids 429 to 652 and 508 to 607, respectively. By immunofluorescence using virus-infected Huh7.5 cells as targets both antibodies were reactive with all of the nine different HCV genotypes/subtypes tested. The antibodies showed a different pattern of immuno-staining; while #37 gave granular reactions mostly located in the periphery of the nucleus, #55 gave diffuse staining throughout the cytoplasm. Both antibodies were shown by immuno-gold electron microscopy to bind to intact viral particles. In a neutralization assay (focus-forming unit reduction using chimeric infectious HCV containing structural proteins derived from genotypes 1a, 1b, 2a, 2b, 3a, 4a, 5a, 6a, and 7a), #55 inhibited the infection of all HCV genotypes tested but genotype 7a to a lesser extent. #37 did not neutralize any of these viruses. As a broadly cross-neutralizing human antibody, #55 may be useful for passive immunotherapy of HCV infection.

## Introduction

Hepatitis C virus (HCV) is a member of the *Flaviviridae* family and contains a 9.6 kb positive-strand RNA genome. The virus has been classified into seven major genotypes. The envelope glycoproteins, E1 and E2, mediate viral entry via cellular co-receptors, including CD81, claudin-1, occludin, and SBR1. The E1 and E2 proteins, located on the surface of viral particles, are the potential targets of neutralizing antibodies. At present, however, neither antibody-based prophylaxis nor an effective vaccine is available.

HCV persists in the presence of circulating antibodies. It has been speculated that this relates to the highly mutable, quasispecies nature of this RNA virus and the continual emergence of neutralization-resistant strains. However, the persistence of HCV in the presence of anti-HCV antibodies can not be fully explained by high variability alone. It has been found that neutralizing activity is detectable in sera from infected patients during both acute and persistent HCV infection [1], [2], and that high titers of neutralizing antibodies correlate with natural resolution of chronic hepatitis C [3]. Further, polyclonal hyper-immune antibodies to the E2 protein have been shown to prevent or delay the onset of HCV infection in chimpanzees when administrated before exposure to the virus [4]. The ability of HCV to persist in its host despite the presence of neutralizing antibodies remains unexplained.

With the advent of recently developed systems to study the full cycle of HCV infection [5], various human monoclonal antibodies to the E1 and E2 proteins have been evaluated for their neutralizing activity and some of them were found to contain broadly cross-neutralizing antibodies [6]–[11]. Passive immunotherapy with such antibodies has preventive and therapeutic potential particularly for preventing HCV re-infection in liver transplant recipients.

During the course of our studies on lymphoblastoid cell lines producing antibodies against HCV, we were able to isolate one clone producing broadly cross-neutralizing antibodies and one clone producing non-neutralizing antibodies from a well-characterized HCV-carrier (patient H). Isolation and characterization of these human monoclonal antibodies are detailed in this report.

## Materials and Methods

### Peripheral Blood Mononuclear Cells (PBMC) and Cell Lines

Following written informed consent, the blood sample was obtained in 2000 from patient H who developed chronic HCV infection after transfusion in 1977 [12]. The work was conducted with approval from the Institutional Review Board of the Clinical Center, National Institutes of Health, Bethesda, USA. (IRB # 91-CC-0117). PBMC were isolated by Ficoll-Isopaque (Pharmacia, Uppsala, Sweden), washed three times in phosphate-buffered saline (PBS), re-suspended in Cell Culture Freezing Medium (Life Technologies Japan, Tokyo, Japan), and stored at –80°C until use. Huh 7 cells, a cell line derived from a hepatocellular carcinoma, and highly permissive Huh7.5 cells [13] (provided by C. Rice, Rockefeller University, USA) were cultured in Dulbecco’s modified Eagle’s medium (DMEM) (Wako, Tokyo, Japan) supplemented with 10% fetal bovine serum (FBS) (Nichirei, Tokyo, Japan). Cells were grown at 37°C in a CO_2_ incubator.

### Immunofluorescence (IF)

After fixation in ice-cold 100% acetone for 5 min, cells were incubated with primary antibody for 30 min at room temperature, washed 3 times in PBS, and incubated with a 1∶200 dilution of the AlexaFluor 488 (Invitrogen, Carlsbad, CA, USA) secondary antibody for 30 min at room temperature. The samples were examined under a TE200 fluorescence microscope (Nikon, Tokyo, Japan).

### Equilibrium Centrifugation in Sucrose Density Gradient (SDG)

A crude supernatant containing HCV was centrifuged at 2,380×g for 15 min at 4°C, filtered through the 0.45 µm membrane, concentrated approximately 100-fold using Amicon Ultra-15 centrifugal filter unit with Ultracel-100 (100 kD cut-off) membrane (Millipore, Billerica, MA, USA). The concentrated sample (1.5 ml) was overlaid on 6 ml of a discontinuous gradient with 10, 20, 30, 40, 50, and 60% (w/v) sucrose steps and centrifuged at 289,000×g for 20 h at 4°C in a CS 100GXL centrifuge (Hitachi, Tokyo, Japan). Buoyant density of fractions was determined by refractometry and expressed in g/ml.

### Immuno-gold Electron Microscopy (EM)

For preparing a concentrated virus sample, fraction 3 obtained from the SDG centrifugation described above was diluted in 6.5 ml PBS and spun down at 215,000×g for 4 h at 4°C in a S58A-0015 rotor (Hitachi, Tokyo, Japan). The resulting pellet was suspended in 50 µl of PBS, mixed with an equal volume of antibody #55, #37, or a control antibody (500 µg/ml), and incubated overnight at 4°C. The mixture was then treated with 10 µl of goat anti-human IgG conjugated with colloidal gold-particles (Jackson Labs, Grove, PA, USA) overnight at 4°C. The sample was placed on a high resolution carbon grid, STEM100Cu (Oken, Tokyo, Japan), negatively stained with 2% uranyl acetate solution, and examined under a JEM-100C transmission electron microscope (JEOL, Tokyo, Japan) at 100 kV.

### Reverse-transcription (RT), PCR, and Quantitative PCR (qPCR)

Extraction of RNA, RT, and PCR were carried out as described previously [14]. The amount of HCV cDNA was measured by qPCR using SYBR Premix Ex Taq (Takara, Tokyo, Japan) with an ABI Prism model Fast 7700 instrument (Applied Biosystems, Tokyo, Japan). To determine copy numbers, standard curves were prepared with serial 10-fold dilutions of a known amount of a plasmid bearing the amplified HCV sequence. We used primers that amplified the 5′ non-coding region of the viral genome. The sequences of the primers used were 5′-TTC ACG CAG AAA GCG TCT AG-3′ as a sense primer and 5′-CCC TAT CAG GCA GTA CCA CA-3′ as an anti-sense primer [15]. For detection of RNA encoding the V_H_ regions of antibodies, we used primer pair CG1z (5′-GCA TGT ACT AGT TTT GTC ACA AGA TTT GGG-3′) and VH6a (5′-CAG GTA CAG CTC GAG CAG TCA GG-3′) for #37, and primer pair CG1z and VH3a (5′-GAG GTG CAG CTC GAG GAG TCT GGG-3′) for #55. The RT-PCR products were cloned into pCR4TOPO (Invitrogen, Carlsbad, CA, USA ) and the molecular clones were sequenced with an ABI PRISM^TM^310 Genetic Analyzer (Applied Biosystems, Tokyo, Japan).

### Expression of HCV E2 Proteins

Forns et al., [16] reported that the HCV E2 protein, when expressed on the cell surface, acquired its native conformation more efficiently when truncated at amino acid (aa) 661 of the viral genome. Therefore, we prepared expression vectors encoding truncated forms of E2 (aa 384 to 661) derived from HCV isolates of patient H, obtained in 1977 (strain H77, AF011751) and in 2000 (strain H00) for the screening of antibody-positive clones. For epitope mapping, we prepared vectors encoding various sizes of E2 proteins derived from strain H77. The inserts were amplified by RT-PCR, cloned into pDisplay (Invitrogen, Carlsbad, CA, USA) in frame between a signal sequence and a trans-membrane domain. All clones were sequenced to ensure that the DNA encoded the authentic HCV sequence. Huh 7 cells were grown in Lab-Tek 8-chamber slides (Nagle Nunc, Naperville, IL, USA) until 80% confluent and transfected with the constructs using SuperFect (Qiagen, Valencia, CA, USA) according to the manufacturer's instructions. After 48 h, cells were washed, fixed with cold acetone for 5 min, and stored at –80°C until use. Expression of the E2 proteins was verified by IF with rabbit hyperimmune sera raised against various domains of HCV E2.

### HCV Plasmids and Generation of Infectious HCV

Plasmid pJFH1 that contains full-length cDNA of HCV strain JFH1 was provided by T. Wakita (National Institute of Infectious Diseases, Tokyo, Japan) [5]. Plasmids pFK-JFH/Con1/C-842-dg, pFK-JFH/J6/C846-dg, and pFK-JFH1/H77/C842-dg to generate chimeric infectious HCV Con1/C3, J6/C3, and H77/C3, respectively, were given by R. Bartenschlager (University of Heidelberg, Heidelberg, Germany) [17]. Plasmids pH77C/JFH1, pJ4/JFH1, pJ6/JFH1, pJ8/JFH1, pS52/JFH1, pED43/JFH1, pSA13/JFH1, pHK6a/JFH1, and pQC69/JFH1 to generate chimeric infectious HCV were provided by J. Bukh (Copenhagen University Hospital, Hvidovre, Denmark) [18]–[22]. These chimeras are JFH1-based recombinants expressing core-NS2 of genotype 1 to 7 isolates. For the synthesis of HCV RNA, the plasmids were transcribed using a Megascript T7 kit (Ambion, Austin, TX, USA). To generate infectious HCV, the *in vitro* transcribed viral genomic RNA was transfected into Huh7.5 cells by electroporation using a Gene Pulser system (Bio-Rad, Hercules, CA, USA) or by using Lipofectamine 2000 reagent (Invitrogen, Carlsbad, CA, USA ) as described by the manufacturer. The culture supernatants collected at 2–7 days after transfection were centrifuged, passed through a 0.45 µm filter, and inoculated into naïve Huh7.5 cells. After additional passages on naïve cells, the cell-free supernatants containing HCV were concentrated approximately 10-fold using Amicon Ultra-15 (Millipore, Billerica, MA, USA) and measured for their infectivity titers. Aliquots were stored at −80°C until use.

### Infectivity Titration

Virus titers were determined by focus-forming units (FFU) assay. Huh7.5 cells were seeded at 2×10^5^ cells per well in 24-well plates and cultured overnight. Test samples were diluted serially 10-fold and each dilution was inoculated into the cells. After incubation for 6 h at 37°C, the cells were supplemented with fresh complete DMEM and cultured for 24 h. The cells were then immuno-stained and HCV-positive foci were manually counted under a fluorescence microscope. Each test was performed in duplicate or triplicate. The virus titer was expressed in FFU per ml sample, as determined by the mean number of IF-positive foci detected in a whole well.

### Virus Neutralization Assays

Neutralization of HCV infection was assessed by the FFU reduction assay. Two independent assays were performed in the different laboratories. The first method was as follows: A 0.5 ml of serial 5-fold dilutions of #37, #55, or an irrelevant control antibody (human-IgG) (Sigma-Aldrich, St. Louis, MO, USA) was pre-incubated at 4°C overnight with an equal volume of the virus solution containing approximately 300 FFU/ml of HCV. The mixtures were inoculated into Huh7.5 cells (5×10^5^/well) cultured on a 15mm-coverglass in 12-well plates. After incubation for 48 h, cells on the glass were fixed with cold 100% acetone and subjected to indirect IF for the detection of infected foci using a serum from patient H followed by the AlexaFluor 488 secondary antibody. IF-positive foci on the whole coverglass were manually counted under a fluorescence microscope. Each test was performed in duplicate. The second method was as follows: A 0.1 ml of the dilutions containing 0.2, 1, 3, 10, 30, 100, 300, or 1000 µg/ml of #55 or a control antibody was pre-incubated at 37°C for 1 h with an equal volume of the virus solution containing 10^2^ or 10^3^ FFU/0.1 ml of HCV. The mixtures were inoculated onto Huh7.5 cells (10^5^ cells/well in 24-well plates). After 3 h of adsorption, the inocula were removed and fresh complete DMEM were added to the wells. At 24 h post-infection, cells were fixed with 4% paraformaldehyde (Wako, Tokyo, Japan) followed by permeabilization with 0.1% Triton-100 (Wako, Tokyo, Japan). The cells were then immuno-stained for the HCV proteins, counterstained with Hoechst 33342 (Invitrogen, Carlsbad, CA, USA), and examined under a BZ-9000 fluorescence microscope (Keyence, Osaka, Japan). The number of HCV infected cells in each well was manually counted. The percent neutralization was calculated as the percent reduction of FFU compared with virus incubated with the control antibody. The NT_50_ value_,_ lowest concentration (µg/ml) of antibody required for 50% reduction of FFU, was determined by curvilinear regression analysis.

## Results

### Establishment of Human Lymphoblastoid Cell Lines Producing Monoclonal Antibodies to the Envelope E2 Protein of HCV

PBMC obtained from patient H were infected with Epstein-Barr virus, strain B95-8, as we described previously [23], and cultured at 37°C in a 75 ml-flask in medium RPMI1640 (Life Technologies Japan, Tokyo, Japan) containing 10% FBS. Ten days later, the cells were distributed in 96-well plates in an amount of 10^4^ cells/0.2 ml/well. After 4 days of cultivation, supernatant from each well was screened for presence of antibodies by IF using Huh 7 cells expressing the E2 protein (aa 384 to 661) derived from HCV strain H77. Cells in the well that gave a positive signal were re-distributed into 96-well plates and the wells were screened again. This procedure was repeated 5 times until all of the tested wells became positive on two successive assays. When cellular RNA was extracted and the V_H_ region of antibody was amplified by RT-PCR, identical sequence was obtained from three randomly selected wells, suggesting that the cells were clones. Antibody from this clone was designated as #37.

**Figure 1:**
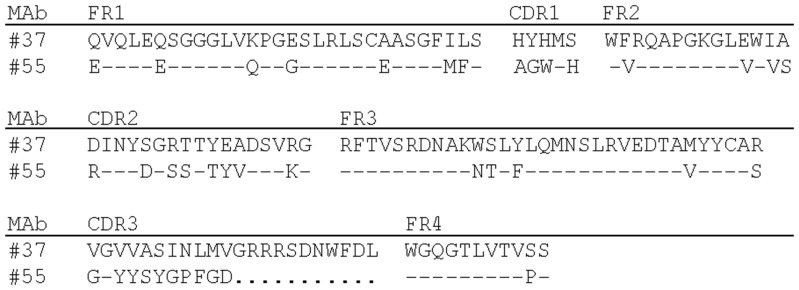
Amino acid sequences of the V_H_ regions of #37 and #55. Residues identical to #37 sequences are indicated by a dash. Dots indicate gaps compared with the sequence of #37. MAb, monoclonal antibody; FR, framework regions; CDR, complementarity-determining regions.

**Figure 2:**
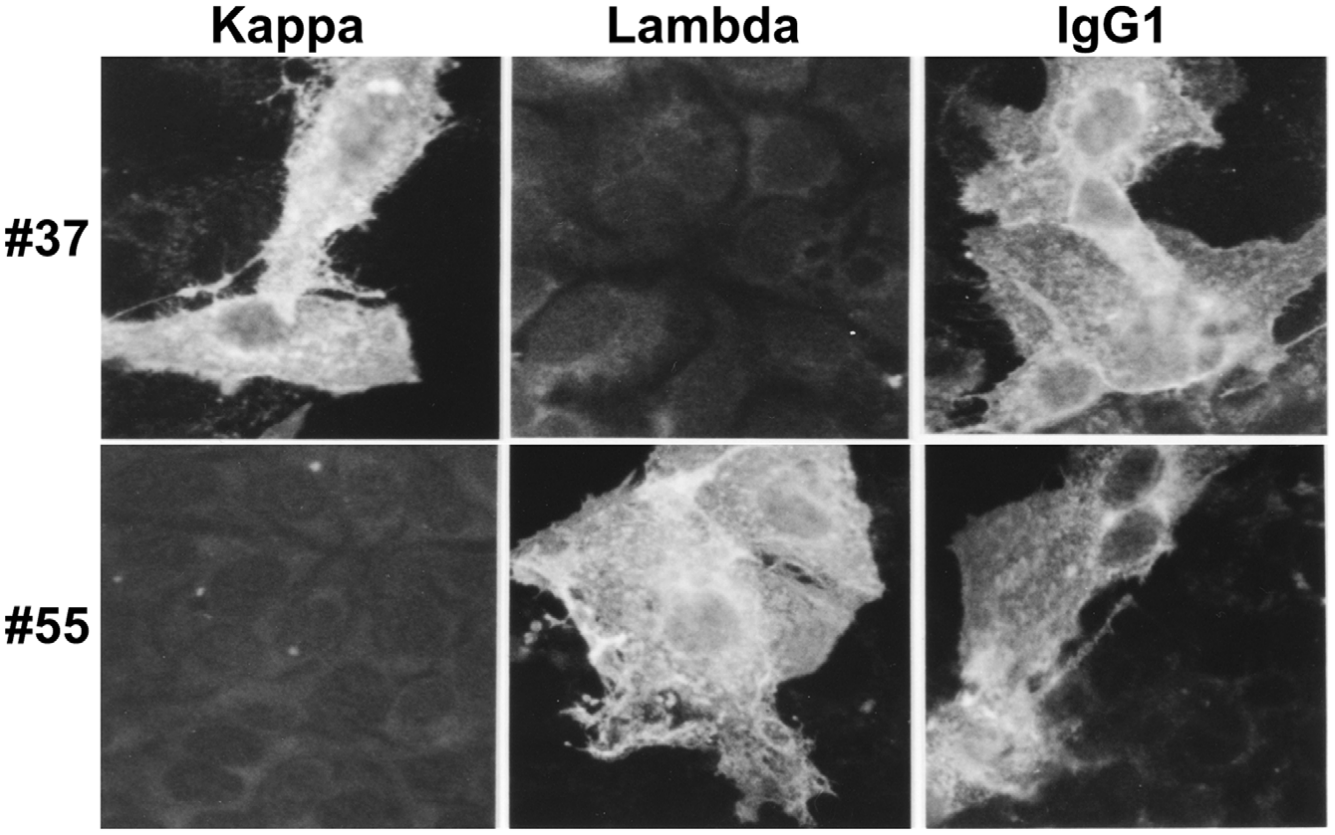
Isotyping revealed that #37 and #55 were IgG1/kappa and IgG1/lambda, respectively. Huh7 cells expressing the HCV E2 (aa 384–661) derived from strain H77 were incubated with #37 or #55, washed, and stained with fluoresceinated anti-human IgG1, anti-human lambda, or anti-human kappa.

With a similar procedure we obtained antibody #55. For the screening of #55, Huh7 cells expressing the E2 protein derived from HCV strain H00 was employed as a target. **Figure 1** shows deduced amino acid sequences of the V_H_ regions for #37 and #55. The antibodies were isotyped by IF with Huh7 cells expressing the E2 protein (aa 384–661) of HCV strain H77, using specific secondary antibodies to human IgM, IgG1, IgG2, IgG3, and IgG4 subclasses, and to lambda and kappa light chains (Binding Site Inc., San Diego, CA, USA). As shown in **Figure 2**, #37 was IgG1/kappa and #55 was IgG1/lambda. The IgG was purified from the supernatants using a HiTrap protein G HP column (GE Healthcare, Uppsala, Sweden) and used for further characterization.

### Epitope Mapping

**Figure 3:**
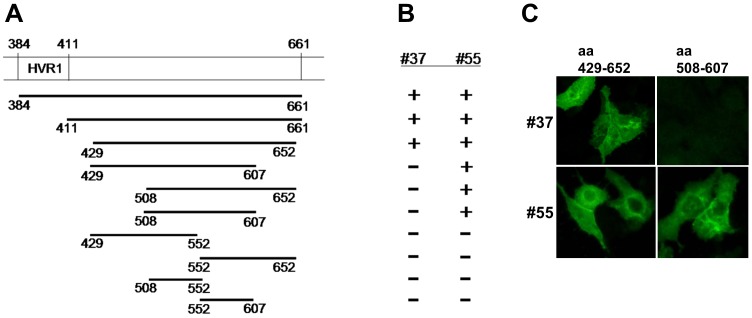
Epitope mapping revealed that #37 and #55 recognized the regions aa 429 to 652 and aa 508 to 607 of the E2 protein, respectively. (**A**). A panel of Huh7 cells expressing various truncations of the E2 protein derived from HCV strain H77 was generated. At the top of the graphic, aa 384 to 661 of the E2 protein is depicted with aa numbers. HVR1, hyper variable region 1. (**B**). Results of the assays for reactivity by IF. +, positive recognition; -, negative recognition. (**C**). IF-reactions of #37 and #55 against the cells expressing aa 429–652 and aa 508–607.

Both #37 and #55 failed to react with the E2 protein in the western blot assay. Therefore, these antibodies were considered to recognize conformational epitopes. In order to map epitope sequences, we prepared expression vectors encoding various regions of the E2 protein derived from HCV strain H77. They include the regions aa 384 to 661, aa 411 to 661, aa 429 to 652, aa 429 to 607, aa 508 to 652, aa 429 to 552, aa 508 to 607, aa 552 to 652, aa 508 to 552, and aa 552 to 607. Huh7 cells expressing these regions were tested by IF for reactivity with #37 and #55. As shown in **Figure 3**, #37 was reactive with the expressed form of the E2 protein containing aa 429 to 652, but not with smaller sizes than this region. In contrast, #55 was reactive with the truncated form down to the region aa 508 to 607. These results indicate that the target epitopes of #37 and #55 are located in the regions of aa 429 to 652 and aa 508 to 607, respectively.

### Cross-reactivity with Different HCV Genotypes and Binding Ability

Recent development of infectious chimeric HCV [17]–[22] has made it possible to investigate cross-genotype reactivity of the antibodies utilizing virus-infected cells as a target. We examined the cross-reactivity of #37 and #55 by IF using Huh7.5 cells infected HCV with different genotype E2 proteins. Genotypes tested were 1a, 1b, 2a, 2b, 3a, 4a, 5a, 6a, and 7a. Both #37 and #55 were reactive by IF with all genotypes tested.

Binding ability of #37 and #55 was assessed by measuring the minimum concentration of the antibodies required for an IF-positive reaction using HCV-infected Huh7.5 cells as targets. The HCV inocula tested were strain JFH1 (genotype 2a) and ten chimeric HCV, including H77C/JFH1 (genotype 1a), J4/JFH1 (genotype 1b), Con1/C3 (genotype 1b), J6/JFH1 (genotype 2a), J8/JFH1 (genotype 2b), S52/JFH1 (genotype 3a), ED43/JFH1 (genotype 4a), SA13/JFH1 (genotype 5a), HK6a/JFH1 (genotype 6a) and QC69/JFH1 (genotype 7a). Antibody solutions containing 5 µg/ml of IgG were two-fold serially-diluted and each dilution was tested by IF for a positive reaction. The results are shown in **Table 1**. The minimum concentration of #55 required was 10–78 ng/ml, while that of #37 was 156–1250 ng/ml except for H77C/JFH1 and S52/JFH1, which required 20 ng/ml. The higher ability of #37 in binding to H77C/JFH1 was possibly because this chimeric virus was a JFH1-based recombinant with homologous envelope E2 of strain H77 from the PBMC-donor. The higher reactivity with S52/JFH1 remains unexplained. Overall, more of #37 was needed for a positive reaction compared to #55, indicating that #55 has a higher binding ability than #37.

**Table 1:** Binding activity measured by immunofluorescence.

Virus (genotype)	Minimum concentration (ng/ml) required for positive reaction	
	#37	#55
H77C/JFH1 (1a)	20	10
J4/JFH1 (1b)	156	78
J6/JFH1 (2a)	1250	20
J8/JFH1 (2b)	1250	78
S52/JFH1 (3a)	20	10
ED43/JFH1 (4a)	625	20
SA13/JFH1 (5a)	156	78
HK6a/JFH1 (6a)	156	78
QC69/JFH1 (7a)	1250	78
Con1/C3 (1b)	625	78
JFH1 (2a)	313	78

### Distribution of Reacting Antigens in the HCV-infected Cells

**Figure 4:**
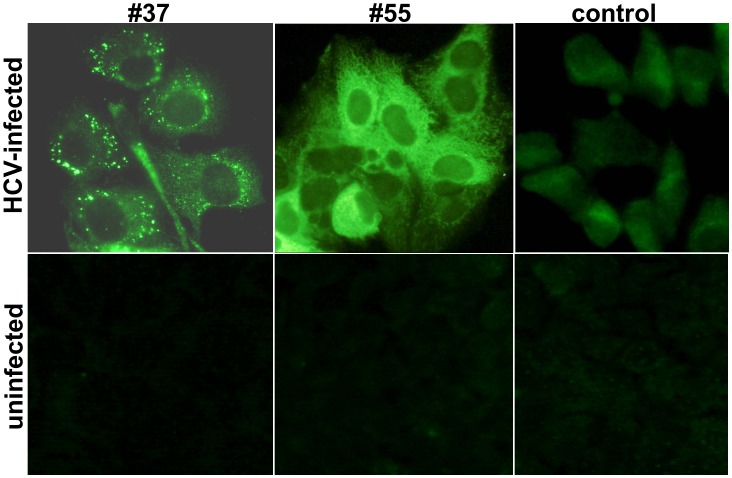
Different pattern of IF-staining by #37 and #55 in the HCV-infected Huh7.5 cells. #37 gave granular IF-reactions scattered in the cytoplasm. #55 gave diffuse staining throughout the cytoplasm. The control antibody (human IgG) gave negative staining. #37, #55, and the control antibody were not reactive with uninfected Huh7.5 cells.

Distribution of the antigens reacting with #55 and #37 in the HCV-infected cells was observed by IF-staining. We examined Huh7.5 cells infected with 12 different inocula of HCV, including strain JFH1 and eleven chimeric HCV with structural proteins derived from various genotypes. **Figure 4** shows IF positive-staining by #37 and #55 observed in the cells infected with strain JFH1. The antibodies produced a different pattern of staining; while #37 gave coarse granular staining mostly located in the periphery of the nucleus, #55 gave diffuse staining throughout the cytoplasm. Similar patterns of IF-staining were observed for other chimeric HCV tested, including H77C/JFH1, J4/JFH1, J6/JFH1, J8/JFH1, S52/JFH1, ED43/JFH1, SA13/JFH1, HK6a/JFH1, QC69/JFH1, H77/C3, and Con1/C3. An irrelevant control antibody (human IgG) did not give such positive staining in the infected cells. #37, #55, and the control antibody were not reactive with non-infected Huh7.5 cells.

### Ability to Recognize HCV Particles

**Figure 5:**
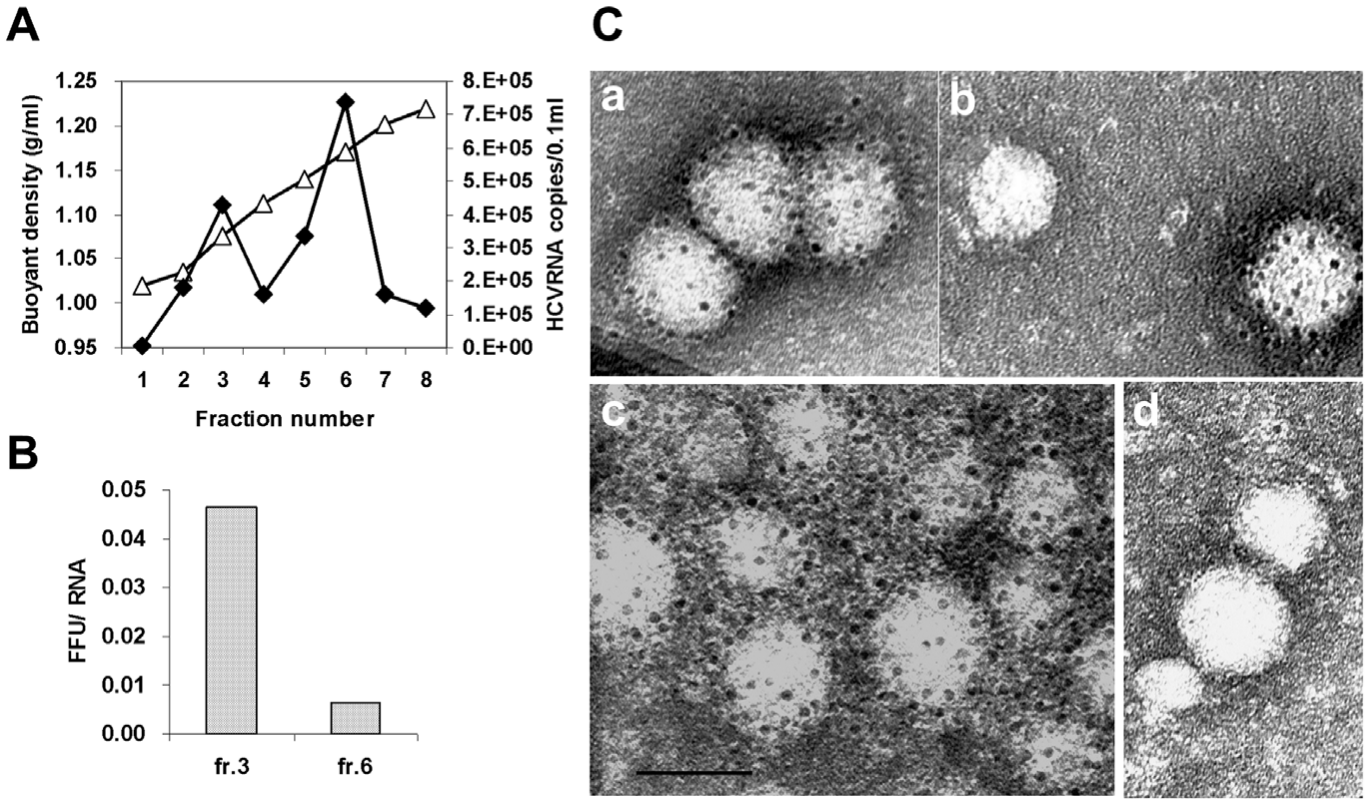
Immuno-gold EM demonstrated that both #37 and #55 recognized HCV intact particles. (**A**). Equilibrium SDG centrifugation. ♦, HCV RNA copies measured by RT-qPCR; Δ, buoyant densities. (**B**). The ratio of infectivity titer (FFU) to HCV RNA copies of fractions 3 and 6. (**C**). Immuno-gold EM. Viral particles in fraction 3 were treated with #37 (**a, b**), #55 (**c**), or a control antibody (**d**) followed by anti-human IgG conjugated with colloidal gold particles, and examined under an electron microscope. HCV-like particles in the sample treated with #37 and #55 were observed with specific labeling of gold particles indicating that the antibodies are capable of binding to viral particles. Bar = 50 nm.

It was recently reported that cell culture-grown HCV particles were pleomorphic, 40–75 nm in diameter, and spherical [24]. To determine whether #37 and #55 are able to recognize intact viral particles, we performed indirect immuno-gold EM using anti-human IgG labeled with colloidal gold particles as a second antibody. As target HCV for this experiment, we employed H77/C3 with homologous envelope E2 of strain H77 from the PBMC-donor. Concentration and purification of viral particles from culture supernatants was carried out by equilibrium SDG centrifugation. **Figure 5A** shows the distribution of HCV RNA measured by RT-qPCR after the centrifugation. Two peaks of viral RNA were obtained at 1.076 g/ml and 1.171 g/ml in fractions 3 and 6, respectively. Copy numbers of HCV RNA were 4.3×10^5/^0.1 ml for fraction 3 and 7.4×10^5/^0.1 ml for fraction 6. These two fractions were further examined by the FFU assay for their infectivity titers. Fractions 3 and 6 had an infectivity titer of 2.0×10^4^ FFU^/^0.1 ml and 4.7×10^3^ FFU/0.1 ml, respectively. Fraction 3 was calculated to have an approximately 9 times higher infectivity titer per HCV RNA than fraction 6 (**Figure 5B**), which was in accordance with our previous observation that the fraction with lower buoyant density was more infectious [25]. Thus, we selected fraction 3 for the EM examination. Fraction 3 was treated with #37 followed by anti-human IgG labeled with colloidal gold-particles, negatively stained, and examined in a transmission electron microscope. We detected HCV-like particles coated with colloidal gold, indicating the binding of #37 to virions. Most of the viral particles reacting with #37 measured approximately 50–60 nm in diameter. **Figure 5C** **(a)** shows an aggregate of three virions coated with specific gold. These viral particles measured approximately 50 nm in diameter. **Figure 5C** **(b)** shows two particles; the one on the right (50-nm in diameter) was coated with colloidal gold, indicating the binding of #37. Another particle on the left (35-nm in diameter) was negative for colloidal gold, indicating that #37 was not reactive with this particle. In addition, the presence of such uncoated particle in the same field suggested that colloidal gold did not bind non-specifically. When fraction 3 was reacted with #55 followed by anti-human IgG labeled with colloidal gold particles, larger aggregates of various-sized viral particles were observed, as shown in **Figure 5C** **(c)**. The viral particles varied in sizes from 40 to 70 nm in diameter. Immuno-gold EM demonstrated that both #37 and #55 can bind to HCV particles. **Figure 5C** **(d)** shows negative reaction of colloidal gold by an irrelevant control antibody (human IgG).

### Neutralizing Activity

**Figure 6:**
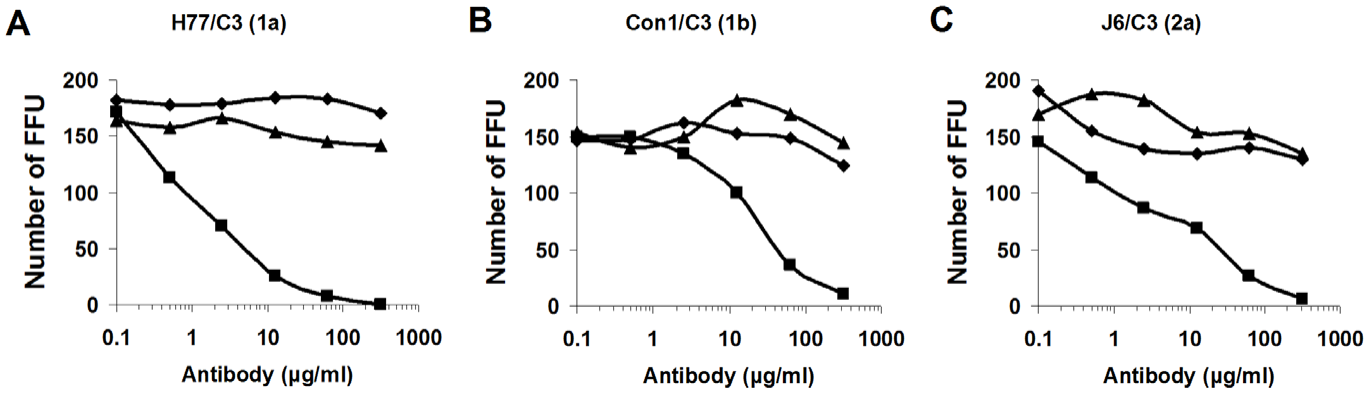
Neutralization assay by FFU reduction. The mean numbers of positive foci are shown for viruses H77/C3 (**A**), Con1/C3 (**B**), and J6/C3 (**C**). Compared to the results obtained with an irrelevant control antibody, #55 inhibited the viral infection in dose-dependent manner for all of the 3 samples tested. Inhibition by #37 was not observed. ♦, control; ▪, #55; ▴, #37.

To investigate whether #37 and #55 could inhibit HCV infection, we performed an *in vitro* neutralization assay by reduction of FFU. As HCV inocula, we used chimeric H77/C3 (genotype 1a), chimeric Con1/C3 (genotype 1b), and chimeric J6/C3 (genotype 2a) in this assay. A virus sample containing approximately 300 FFU/ml of HCV was pretreated at 4°C overnight with #37, #55, or an irrelevant control antibody at a final concentration of 0.1, 0.5, 2.5, 12.5, 62.5, or 312.5 µg/ml and the mixtures were then inoculated into Huh7.5 cells. After 48 h post-infection, IF-positive foci were manually counted under a fluorescence microscope. Each test was performed in duplicate. As shown in **Figure 6**, compared to the results obtained with an irrelevant control antibody, #55 inhibited the viral infection in dose-dependent manner for all of the 3 samples tested. Inhibition by #37 was not observed.

Since #55 was found to have a neutralizing activity as shown above, further examination by the FFU reduction assay was conducted with HCV strain JFH1 (genotype 2) and various chimeric HCV containing the E2 proteins from 9 different genotypes. Chimeric viruses tested included H77C/JFH1 (genotype 1a), J4/JFH1 (genotype 1b), J6/JFH1 (genotype 2a), J8/JFH1 (genotype 2b), S52/JFH1 (genotype 3a), ED43/JFH1 (genotype 4a), SA13/JFH1 (genotype 5a), HK6a/JFH1 (genotype 6a), and QC69/JFH1 (genotype 7a). Two different concentrations of target HCV were tested in the assays. The one virus-sample contained 10^2^ FFU/0.1 ml (no.1) and another 10^3 ^FFU/0.1 ml (no.2). **Table 2** shows the 50% neutralization titers (NT_50_) of #55, a lowest concentration (µg/ml) required for 50% reduction of FFU, calculated by curvilinear regression analysis. #55 neutralized HCV infection of various genotypes (1a, 1b, 2a, 3a, 4a, 5a, and 6a), with the NT_50_ titers ranging from 2 to 127 µg/ml for no.1 and 6 to 231 µg/ml for no.2. Neutralization of genotype 7a (QC69/JFH1) by #55 was less, with a NT_50_ titer of 219 µg/ml for no.1 and >500 µg/ml for no.2.

**Table 2:** 50% neutralization titers (NT_50_) of #55 by FFU reduction.

Virus (genotype)	NT_50_ (µg/ml)*	
	no.1	no.2
H77C/JFH1 (1a)	10.6	10.3
J4/JFH1 (1b)	ND	9.3
J6/JFH1 (2a)	126.6	99.1
J8/JFH1 (2b)	21.1	24.1
S52/JFH1 (3a)	73.0	230.8
ED43/JFH1 (4a)	1.3	7.4
SA13/JFH1 (5a)	5.0	5.7
HK6a/JFH1 (6a)	5.2	7.3
QC69/JFH1 (7a)	218.5	>500
JFH1 (2a)	2.0	ND

* , calculated by curvilinear regression analysis; ND, not done.

### Blocking of Viral Adsorption

**Figure 7:**
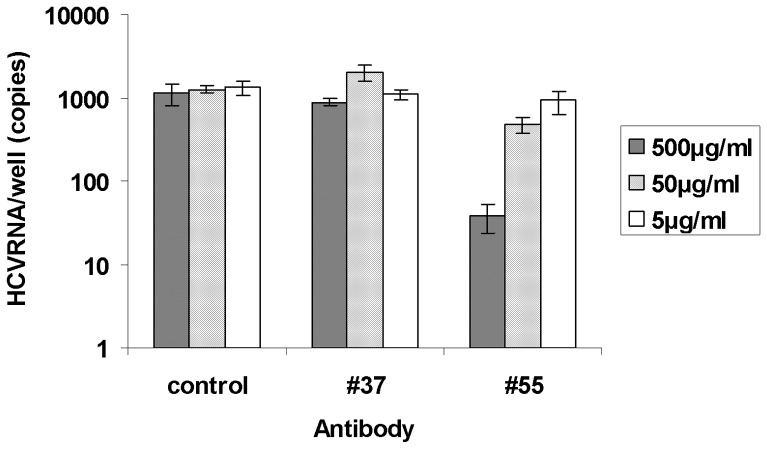
Blocking of viral adsorption by #55. Blocking of viral adsorption measured by RT-qPCR. Compared to the control antibody (human IgG), #55 inhibited viral adsorption in dose-dependent manner. Inhibition by #37 was not observed but rather slightly enhanced at a concentration of 50 µg/ml.

We examined whether #55 blocked viral adsorption to cells by measuring the amount of cell-attached HCV RNA using RT-qPCR. A half ml of the culture supernatant containing 10^5^ FFU/ml of chimeric HCV, H77/C3, was pre-treated at 4°C for 24 h with an equal volume of #55, #37 or a control antibody at a final concentration of 500, 50, or 5 µg/ml. The mixtures were then inoculated onto Huh7.5 cells seeded in 12-well plates (5×10^5^ cells/well). After incubation for 4 h at 37°C, cells were washed 3 times with PBS. Amount of cell-associated HCV RNA in a well was measured by RT-qPCR. Each test was performed in duplicate. Compared to the control antibody (human IgG), #55 inhibited viral adsorption in dose-dependent manner, as shown in **Figure 7**. Inhibition by #37 was not observed but rather slightly enhanced at a concentration of 50 µg/ml.

**Figure 8:**
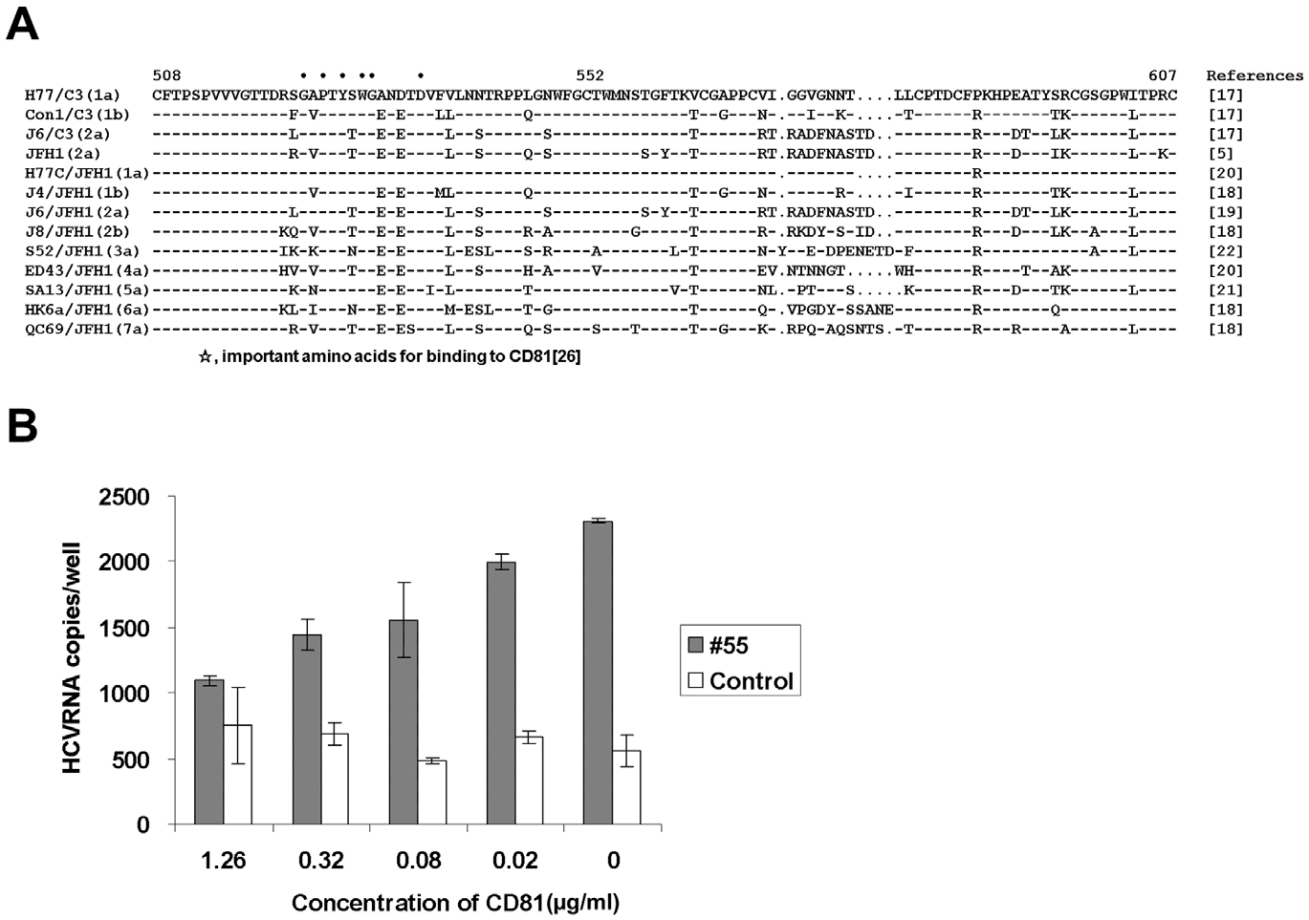
Binding of HCV to #55 was inhibited by soluble recombinant CD81. (**A**). Sequence alignment of aa 508 to 607, the epitope of #55, of various genotypes of HCV employed in the present study. Residues identical to the sequences of H77/C3 are indicated by a dash. Dots indicate gaps. (**B**). H77/C3 virus was treated with various dilutions of soluble CD81.The mixtures were inoculated into a 48-well plate that was pre-coated with #55 or the control antibody. Amount of bound HCV RNA was measured by RT-qPCR.

Specific amino acids (W420, Y527, W529, G530, and D535) in the E2 envelope protein of HCV were reported to be critical for binding to CD81, a principal cellular receptor and they were conserved across all genotypes [26]. As the epitope of #55 includes these amino acid residues, it was possible that #55 blocked virus adsorption by competing with CD81 for a binding site on the E2 envelope. **Figure 8A** shows sequence alignment of aa 508 to 607, the epitope of #55, of HCV employed in the present study. The epitope of #55 contains the residues important for binding to CD81 (asterisks). Thus, we investigated this possibility by testing whether CD81 inhibits binding of HCV to #55 utilizing an assay based on antibody-captured RT-qPCR. A 100 µl of the virus solution containing 10^4^ FFU/ml of H77/C3 was incubated with an equal volume of various dilutions of soluble recombinant human CD81 protein (Origene, Rockville, MD, USA) for 2 h at room temperature. Each mixture was then inoculated into a 48-well plate which was pre-coated with #55 or an irrelevant control antibody (human IgG) at a concentration of 10 µg/ml. The plate was incubated at 4°C overnight. After washing, bound HCV RNA was extracted and quantified by RT-qPCR. As shown in **Figure 8B**, CD81 inhibited the binding of virus to #55 in a dose-dependent manner.

## Discussion

In this study, as an approach to obtain human B cell lines producing antibodies to HCV envelope E2, we applied the EBV transformation method, which is based on the fact that EBV transforms B-lymphocytes of humans *in vitro* into lymphoblastoid cells that synthesize and secrete immunoglobulins. From PBMC collected from a patient persistently infected with HCV strain H (genotype 1a) we have successfully isolated two clones producing anti-HCV E2 antibodies, #37 and #55. At the first screening of culture supernatants, several wells of a 96-well plate were found positive for anti-HCV E2 antibodies. However, most of them became negative as further cultured. Finally #37 and #55 remained as stably producing clones.

There was remarkable contrast between these two antibodies in their properties: (1) #55 appeared to be a broadly cross-neutralizing antibody. In the neutralization assay by FFU reduction, it inhibited infection by HCV genotypes 1a, 1b, 2a, 2b, 3a, 4a, 5a, 6a, and, to a lesser extent, 7a. In contrast, #37 did not neutralize any of the viruses tested. Interestingly it tended to enhance the infection at low concentrations (**Figure 6B and C** **,** and **Figure 7**): (2) the epitope of #55 was mapped to the region of aa 508 to 607 and that of #37 was mapped to the longer region spanning aa 429 to 652 of the E2 protein. #55 seemed unique for broadly cross-neutralizing antibody to have a relatively short conformational epitope, since it has been reported that conformational epitopes reacting with such antibodies are usually retained in the full length E2 [7], [9]: (3) when we tested their cross-reactivity using transfected Huh7 cells expressing the E2 proteins, #37 was reactive with genotype 1a but reacted very weakly with the others, while #55 was broadly reactive with all genotypes tested. However, when examined using the virus-infected cells as targets, #37 was reactive with all HCV genotypes tested, although its binding activity measured by IF was less than that of #55 except for H77C/JFH1(1a) and S52/JFH(3a): (4) in immuno-gold EM, viral particles recognized by #37 were rather homogenous in size and measured approximately 50–60 nm in diameter. On the other hand, #55 produced larger aggregates of various-sized viral particles, probably because of its higher binding activity: (5) the antibodies showed a different pattern of IF-staining in the HCV-infected cells. While #37 gave granular reactions mostly in the periphery of nuclei, #55 gave diffuse staining throughout the cytoplasm (**Figure 4**). The nature of the antigens reacting with #37 and #55 remains to be studied.

Recently, Keck et al. reported that the region aa 529 to 535 of the E2 envelope protein is a CD81 binding region that does not tolerate neutralization escape mutations [27]. The epitope of #55 includes the above mentioned region and #55 blocked virus adsorption by competing with CD 81 for a binding site on the E2 envelope. As #55 is broadly neutralization cross-reactive, it may be very useful in preventing infection by HCV of various genotypes. Sasayama et al. reported that blocking N-glycosylation of aa 534 (aa 532 of strain H77) in this region by substituting asparagine with histidine markedly enhanced the sensitivity of the virus to neutralizing antibodies and suggested that the aa 529 to 535 region is usually protected from the antibody’s access by the N-glycosylation [28]. It is possible that #55 may evade the N-glycosylation mediated protective mechanism of HCV.

A cross-neutralizing monoclonal antibody that could be generated in large volume might be particularly beneficial to prevent the almost universal occurrence of HCV re-infection of transplanted livers and could play other roles in immunoprophylaxis until such time as an effective HCV vaccine is developed and commercialized.
